# D-ribose is elevated in T1DM patients and can be involved in the onset of encephalopathy

**DOI:** 10.18632/aging.102089

**Published:** 2019-07-15

**Authors:** Lexiang Yu, Yao Chen, Yong Xu, Tao He, Yan Wei, Rongqiao He

**Affiliations:** 1 School of Basic Medical Sciences of Southwest Medical University, Luzhou 646000, China; 2 State Key Laboratory of Brain and Cognitive Science, Institute of Biophysics, University of Chinese Academy of Sciences, Beijing 100101, China; 3 Alzheimer’s Disease Center, Beijing Institute for Brain Disorders, Center for Brain Disorders Research, Capital Medical University, Beijing 100069, China; 4 CAS Key Laboratory of Mental Health, Institute of Psychology, Chinese Academy of Sciences, Beijing 100101, China; 5 Affiliated Hospital of Southwest Medical University, Luzhou 646000, China

**Keywords:** D-ribose, benfotiamine (BTMP), cognitive impairment, type 1 diabetic encephalopathy, type 1 diabetes mellitus (T1DM), diabetic encephalopathy

## Abstract

Although many mechanisms have been proposed for diabetic encephalopathy in type 2 diabetes mellitus (T2DM), the risk factors for cognitive impairment in type 1 diabetes mellitus (T1DM) are less clear. Here, we show that streptozotocin (STZ)-induced T1DM rats showed cognitive impairment in both Y maze and Morris water maze assays, accompanied with D-ribose was significantly increased in blood and urine, in addition to D-glucose. Furthermore, advanced glycation end products (AGE), Tau hyperphosphorylation and neuronal death in the hippocampal CA4/DG region were detected in T1DM rats. The expression and activity of transketolase (TKT), an important enzyme in the pentose shunt, were decreased in the brain, indicating that TKT may be involved in D-ribose metabolism in T1DM. Support for these change was demonstrated by the activation of TKT with benfotiamine (BTMP) treatment. Decreased D-ribose levels but not D-glucose levels; markedly reduced AGE accumulation, Tau hyperphosphorylation, and neuronal death; and improved cognitive ability in T1DM rats were shown after BTMP administration. In clinical investigation, T1DM patients had high D-ribose levels in both urine and serum. Our work suggests that D-ribose is involved in the cognitive impairment in T1DM and may provide a potentially novel target for treating diabetic encephalopathy.

## INTRODUCTION

Type 1 diabetes mellitus (T1DM) is a D-glucose metabolic disorder characterized by autoimmune destruction of pancreatic β-cells, leading to insulin deficiency and hyperglycaemia [1]. T1DM can affect different organs and result in many complications; among these complications, diabetic encephalopathy is diabetes-induced brain damage [2]. As early as 1922, diabetes was recognized to lead to cognitive dysfunction [3]. Because an increasing number of people are diagnosed with T1DM or type 2 diabetes mellitus (T2DM), diabetic encephalopathy has become widely recognized [4–6]. Patients with diabetic encephalopathy show both mental and physical symptoms, including an altered mental state, cognitive decline, memory lapses, and changes in personality [2, 7]. Compared with people without diabetes mellitus, people with diabetes are at higher risk of cognitive decline and dementia, such as Alzheimer's disease [8].

Although many pathomechanisms, such as alterations in the vascular supply of the brain and the interaction between insulin and the brain [9], have been proposed for diabetes mellitus, the most attention has been paid to T2DM in ageing people [10]. Unlike T2DM, T1DM is one of the most frequent chronic diseases in children and can start at any age. Some children with T1DM may be at high risk of cognitive deficits, especially those diagnosed at earlier ages [11, 12]. Middle-aged and older adults are also at increased risk for cognitive decline [13, 14]; however, the risk factors for cognitive decline in adults with T1DM have remained unclear thus far [15]. Therefore, identifying the risk factors of T1DM-related cognitive impairment is important.

D-glucose is a long-standing major molecular biomarker for both T1DM and T2DM. However, recent studies have shown that in addition to D-glucose, D-ribose plays a role in T2DM [16, 17]. As a key component of intracorporal biomolecules, including RNA, ATP and riboflavin, D-ribose participates in numerous biochemical processes [18–20] and has an active role in the glycation of protein, producing advanced glycation end products (AGE) [21, 22]. In particular, D-ribose is an important contributor to glycated haemoglobin (HbA1c) [17] and glycated serum protein [23], showing the linkage between aldopentose and diabetes. Intraperitoneal injection with D-ribose (3.2 g/kg.bw, once daily) elicited a significant increase in triglyceride in Sprague-Dawley (SD) rat liver [24]. As described by the European Food Safety Authority [25], toxicological effects could not be ruled out, although the use of D-ribose in nutritional supplement is considered acceptable. Especially in cognitive impairment, ribosylation-induced Tau protein aggregation is highly cytotoxic to neuronal cells [26]. Long-term gavage of D-ribose can also cause cognitive impairment in mice [27]. Increasing evidence suggests that D-ribose is closely related to T2DM and even diabetic complications. However, the relationship between D-ribose and cognitive impairment in T1DM has not yet been investigated.

The streptozotocin (STZ)-injected rats is a common animal model used in T1DM studies [28, 29]. T1DM is associated with neurocognitive dysfunction and astrogliosis [30]. Furthermore, metabolic analyses revealed that T1DM mainly affects metabolic pathways involved in mitochondrial energy failure and impairs the antioxidative system [31]. In the present study, we observed that T1DM patients had abnormally increased levels of D-ribose in blood and urine. STZ-induced T1DM rats exhibited cognitive impairment and had high levels of D-ribose in blood and urine, along with AGE formation, Tau hyperphosphorylation and neuronal death. Transketolase (TKT) was demonstrated to be an important enzyme in regulating D-ribose metabolism in T1DM-related encephalopathy rats. Interestingly, elevation of TKT by benfotiamine (BTMP) rescued D-ribose dysmetabolism, followed by decreases in AGE accumulation, Tau hyperphosphorylation, and neuronal death, as well as the rescue of cognitive impairment in T1DM rats.

## RESULTS

### High D-ribose levels in type 1 diabetic rats

To investigate D-ribose dysmetabolism in T1DM, we prepared a T1DM animal model by administering a single intraperitoneal injection of STZ (n = 30) to SD rats as previously described [32]. Rats injected with citrate buffer (pH 4.2-4.5) were employed as controls (n = 10). Rats were maintained for 10 weeks; during this period, their fasting blood glucose (FBG), body weight and forepaw tensions were monitored every other week (Supplementary Figure 1A–1C). FBG levels in diabetic rats markedly increased after STZ injection, while body weight and tension decreased. Low levels of serum C-peptide, glucagon and insulin and brain insulin were also detected in diabetic rats (Supplementary Figure 1D). These data conformed to the requirements for using rats as a diabetic model, which was observed as D-glucose dysmetabolism.

**Figure 1:**
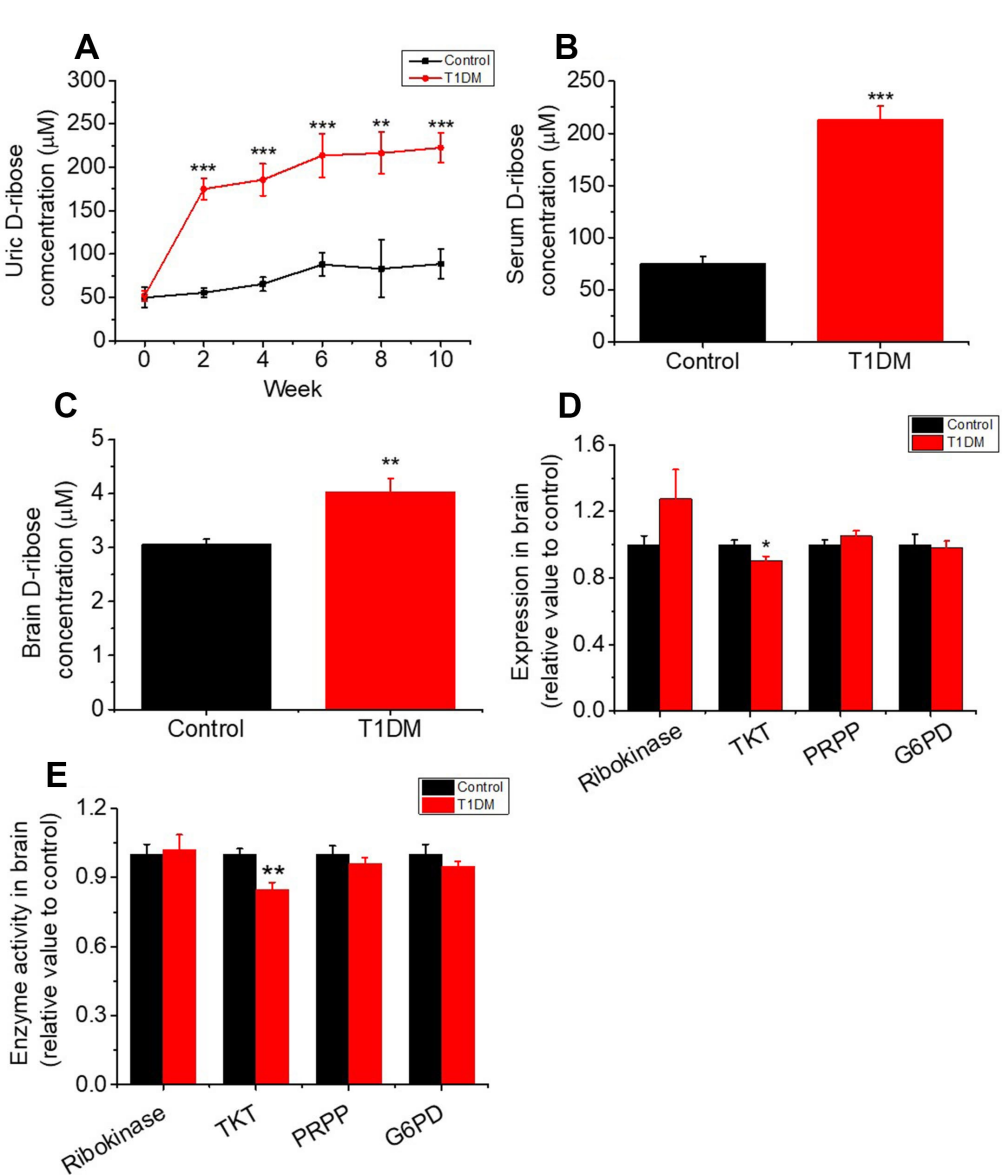
**Increase in the levels of D-ribose and related enzymes in type 1 diabetic rats.** Male SD rats (6–8 weeks) were intraperitoneally injected with STZ (70 mg/kg bw, n=30) and maintained for 10 weeks. Rats injected with saline were used as controls (n=10). Levels of D-ribose in urine were measured at different time intervals (panel **A**). D-ribose levels in serum (panel **B**) and the brain (panel **C**) were determined within 3 days after dissection. The expression and activity levels of ribokinase, transketolase (TKT), 5-phosphoribosyl 1-pyrophosphate (PRPP) and glucose-6 phosphate dehydrogenase (G6PD) in the brain were measured with ELISA kits (panel **D** and **E**). All values are expressed as the mean ± S.E.M. *, *P* < 0.05; **, *P* < 0.01; ***, *P* < 0.001.

To investigate whether D-ribose dysmetabolism occurs in T1DM, we monitored urine D-ribose levels every other week. As shown in Figure 1A, the concentrations of urine D-ribose in diabetic rats were significantly higher than those in control rats (*P* < 0.001). Moreover, serum and brain D-ribose levels were markedly increased in T1DM rats (*P* < 0.001 and *P* < 0.01, Figure 1B and 1C, respectively). Together with the results above, these results indicated that T1DM rats exhibit D-glucose and D-ribose dysmetabolism. However, further investigation was needed to determine why D-ribose levels were increased in T1DM.

### Increase in D-ribose attributed to the inactivation of TKT in T1DM

TKT is a key enzyme in the nonoxidative branch of the pentose phosphate pathway (PPP) that is involved in the metabolism of D-ribose derivatives [33, 34]. To investigate the mechanism of the D-ribose metabolic disorder in T1DM, we measured TKT expression and activity by ELISA. As shown in Figure 1D and 1E, the expression and activity level of TKT were decreased remarkably in T1DM brain tissue. Other kinases, such as ribokinase, D-glucose-6-phosphate dehydrogenase (G6PD) and ribose phosphate pyrophosphokinase (PRPP), which also play roles in regulating D-ribose metabolism, did not show significant changes in expression or activity level in T1DM rats compared to control rats.

**Figure 2:**
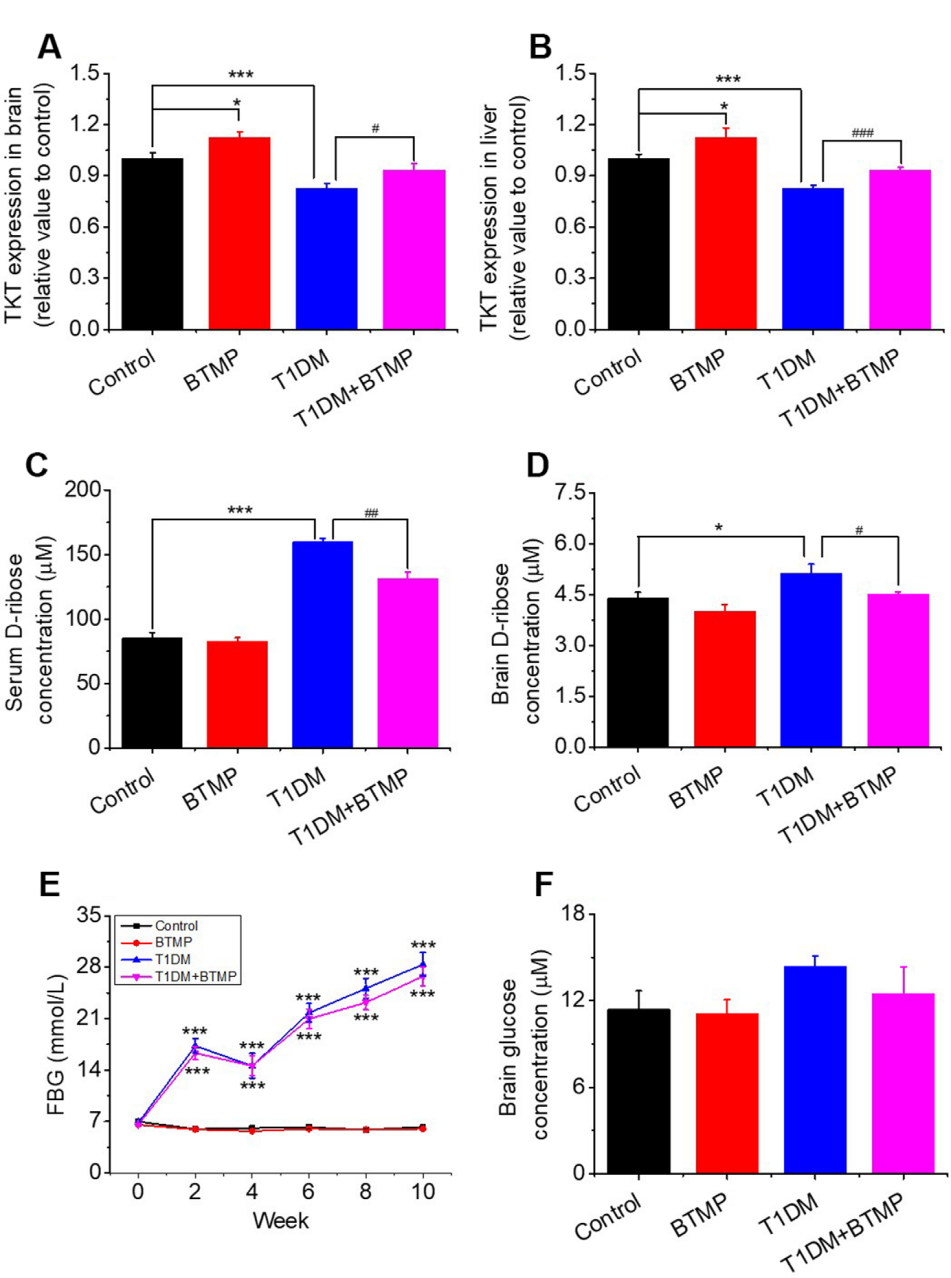
**Effect of benfotiamine (BTMP) on the levels of D-ribose, D-glucose and TKT in T1DM rats.** Conditions for the preparation of T1DM rats are shown in Figure 1. Male rats (6-8 weeks) were divided into four groups as follows: T1DM rats were gavaged with benfotiamine (BTMP, 300 mg/kg bw, once daily) dissolved in carboxymethylcellulose (CMC) (90) (T1DM+BTMP, n=20); T1DM rats were gavaged with CMC (T1DM, n=20); normal SD rats were gavaged with CMC (Control, n=10) and BTMP (n=10) as negative and positive controls, respectively. The expression levels of transketolase (TKT) in the brain (panel **A**) and liver (panel **B**) were measured with ELISA kits. After 10 weeks of domestication, D-ribose levels in the serum (panel **C**) and brain (panel **D**) of rats were measured, and D-glucose levels were measured in the brain (panel **F**). Fasting blood glucose (FBG) was measured every other week (panel **E**). “*” compared to the control group. “#” represents the difference between the T1DM and T1DM+BTMP groups. All values are expressed as the mean ± S.E.M. *, *P* < 0.05; ***, *P* < 0.001; #, *P* < 0.05; ##, *P* < 0.01; ###, *P* < 0.001.

To demonstrate that TKT is linked to D-ribose dysmetabolism, we used BTMP to rescue the TKT change in T1DM rats since BTMP can increase the level of thiamine diphosphate and enhances TKT activity [35]. Diabetic rats (n = 20) were gavaged with BTMP, while the control rats (n = 10) were gavaged with carboxymethylcellulose (CMC) as described previously [17]. The results of the liver and kidney assays after BTMP treatment are shown in Supplementary Table 1. As shown in Figure 2A, administration of BTMP increased brain TKT levels in both normal rats (*P* < 0.05) and diabetic rats (*P* < 0.05). As expected, a similar result showing that BTMP rescued the expression of TKT was observed in the liver in T1DM rats compared with that in control rats (Figure 2B). Both the activity and expression (Western blots) of TKT in the liver and brain were significantly rescued after BTMP administration (Supplementary Figure 2). By contrast, D-ribose levels in both serum and brain were significantly decreased after BTMP administration (Figure 2C, 2D). However, brain D-glucose levels showed no marked differences (*P* > 0.05) between T1DM rats and BTMP-gavaged T1DM rats (Figure 2E). Under the experimental conditions, BTMP did not rescue or decrease FBG levels in T1DM rats (Figure 2F). However, BTMP could partially rescue the body weights of T1DM rats but not the forepaw tension or insulin levels in the brain and serum (Supplementary Figure 3). That is, administration of BTMP can regulate the metabolism of D-ribose rather than D-glucose in rats via activation of TKT.

### Gavage of BTMP ameliorates cognitive impairment in T1DM rats

According to McCrimmon and colleagues, both T1DM and T2DM are related to cognitive dysfunction [36]. Here, we investigated whether T1DM rats experienced cognitive impairment. First, compared with control rats, T1DM rats showed significantly fewer correct alterations in the Y maze test (Supplementary Figure 4A). In the Morris water maze test, the escape latency in the training session was significantly longer among T1DM rats (Supplementary Figure 4B), and the percentage of time spent in the target quadrant in the probe trial was markedly lower for T1DM rats (Supplementary Figure 4C). T1DM rats also showed fewer platform crossings than did control rats, but the difference was nonsignificant (Supplementary Figure 4D). Representative images of the performance path of the rats are shown in Supplementary Figure 4E. These results indicated that T1DM rats exhibited cognitive impairment, which was regarded as type 1 diabetic encephalopathy. In addition, rats with type 1 diabetic encephalopathy also showed anxiety behavior based on open field and elevated plus maze assays (Supplementary Figure 5).

**Figure 3:**
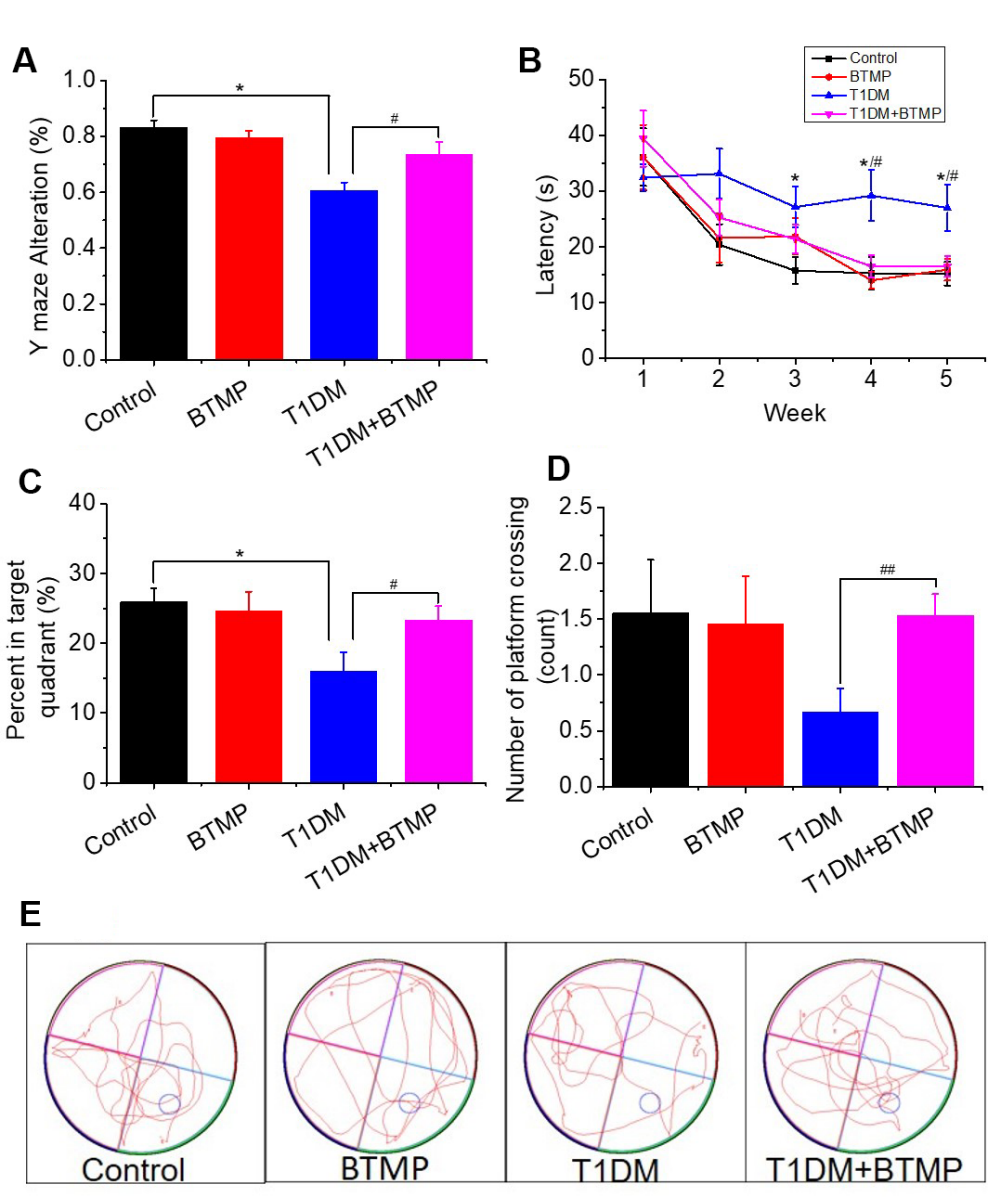
**Rescue of spatial learning and memory abilities in T1DM rats with BTMP.** Animal groups and treatments were as described in Figure 2 except that rats were subjected to Y maze and Morris water maze tests. The accuracy of Y maze alternation was detected (panel A). The escape latency (panel B), percentage of time spent in the target quadrant (panel C) and number of platform crossings (panel D) were recorded. Representative images of the performance path are shown (panel E). “*” represents the difference between the Control and T1DM groups. “#” represents the difference between the T1DM and T1DM+benfotiamine (BTMP) groups. The number of those groups are control (n=10), BTMP (n=9), T1DM (n=15) and T1DM+BTMP (n=15). All values are expressed as the mean ± S.E.M. *, *P* < 0.05; #, *P* < 0.05; ##, *P* < 0.01.

To demonstrate whether cognitive impairment in T1DM rats was linked to D-ribose dysmetabolism, we tested the cognitive ability of T1DM rats with BTMP gavage. In the Y maze test, BTMP-gavaged T1DM rats exhibited significantly more correct alterations than in T1DM rats without BTMP gavage (Figure 3A). In the Morris water maze test, T1DM rats gavaged with BTMP spent less time searching for the platform than did T1DM rats without BTMP gavage (Figure 3B). After platform withdrawal, the time spent in the target quadrant and the number of platform crossings were significantly higher in the T1DM group gavaged with BTMP than in that without BTMP gavage (Figure 3C, 3D). Representative images of the rats’ performance paths are shown in Figure 3E. These data suggested that the alleviation of cognitive impairment by treatment with BTMP is related to a decrease in D-ribose in STZ-induced T1DM rats.

### D-ribose is linked to AGE, Tau hyperphosphorylation and neuronal death

As hyperphosphorylated Tau and the resultant neurofibrillary tangles and AGE are closely related to cognitive impairment [37, 38], and high dose D-ribose treatment resulted in AGE aggregation and Tau hyperphosphrylation [39]. We wondered whether cognition-impaired T1DM rats exhibit Tau hyperphosphorylation and AGE accumulation along with a decrease in D-ribose. As shown, Tau was remarkably hyperphosphorylated at Ser396, AT8 (Ser199 and Ser202), while nonphosphorylated Tau (recognized by anti-Tau-1 antibody) was decreased in both the cortex (Supplementary Figure 6A, a’) and hippocampus (Supplementary Figure 6B, b’) in T1DM rats. At the same time, markedly high AGE levels were detected in both the cortex and hippocampus. These data suggest that T1DM rats suffer from Tau hyperphosphorylation as well as AGE accumulation in the brain.

To investigate whether D-ribose dysmetabolism is linked to Tau hyperphosphorylation and AGE accumulation, we gavaged T1DM rats with BTMP and measured Tau phosphorylation and AGE levels. As shown in Supplementary Figure 7, AGE in both the cortex and hippocampus in BTMP-gavaged T1DM rats were significantly decreased compared with those without BTMP gavage. Furthermore, the results from the glycated serum protein (GSP) assay showed a marked decrease in GSP in T1DM rats after treatment with BTMP (Supplementary Figure 7C). These data demonstrated that D-ribose plays a role in AGE accumulation in T1DM rats.

Along with the distinct decrease in AGE accumulation, Tau hyperphosphorylation was also reduced by BTMP administration. Tau phosphorylation levels (AT8 and pSer396) in the cortex and hippocampus were significantly reduced, while nonphosphorylated Tau levels (Tau-1) were increased in T1DM rats treated with BTMP compared with control rats (Supplementary Figure 7). That is, Tau hyperphosphorylation is related to the D-ribose dysmetabolism in T1DM rats.

**Figure 4:**
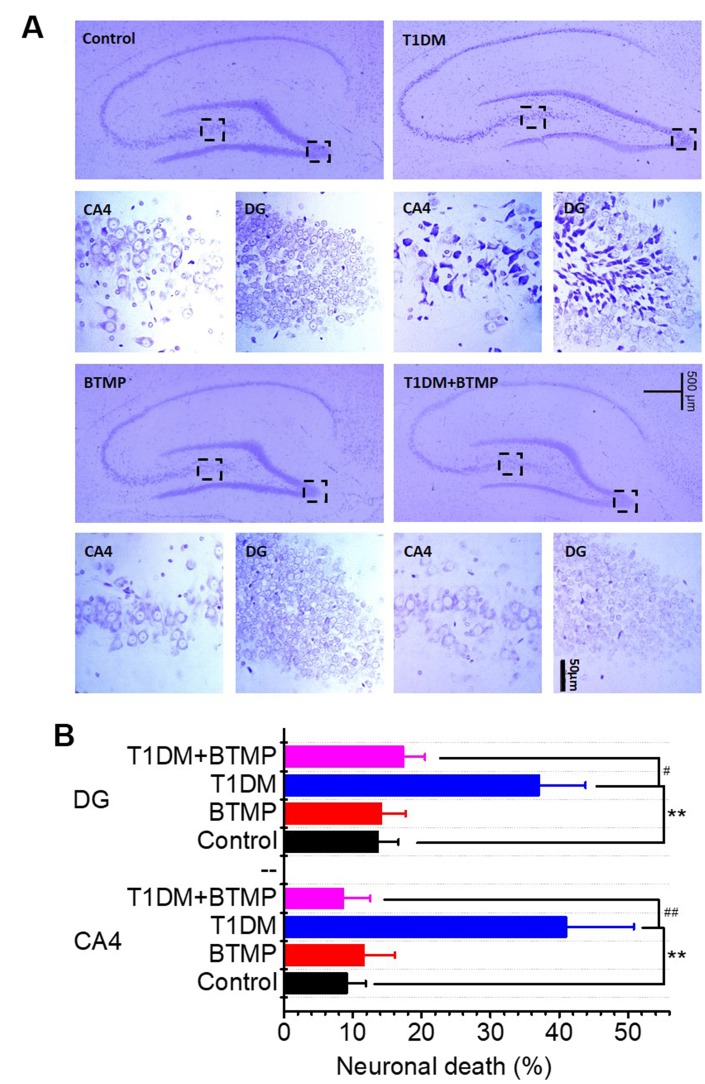
**Nissl staining of hippocampal neurons of rats treated with BTMP.** Animal groups and treatment were as described in Figure 2 except that hippocampal slices were prepared and stained with cresyl violet (panel **A**). Numbers of necrotic neurons were counted under a microscope as described in the Materials and Methods (panel **B**). The number of those groups are control (n=10), BTMP (n=9), T1DM (n=10) and T1DM+BTMP (n=10). All values are expressed as the mean ± S.E.M. “*” compared to the control group. “#” represents the difference between the T1DM and T1DM+BTMP groups. All values are expressed as the mean ± S.E.M. **, *P* < 0.01; #, *P* < 0.05; ##, *P* < 0.01.

Neuronal loss is regarded as the most important pathological feature of age-related cognitive impairment. We performed immunochemical experiments to determine neuronal death in the brains of T1DM rats. As shown in Figure 4A, neuronal death was distinctly observed in the hippocampal CA4/DG area of T1DM rats. However, neuronal death in the brains of T1DM rats was greatly reduced by treatment with BTMP (Figure 4A, 4B). The decrease in D-ribose induced by BTMP occurred with the amelioration of pathological features, such as AGE accumulation, Tau hyperphosphorylation and neuronal death. These data suggested that D-ribose dysmetabolism is closely related to cognitive impairment and that BTMP can be used as a potential medicine to rescue cognitive impairment via a decrease in D-ribose levels. Additionally, the anxious behavior was observably improved after BTMP administration in the open field and elevated plus maze assays (Supplementary Figure 8).

### Increased D-ribose levels in T1DM patients

**Figure 5:**
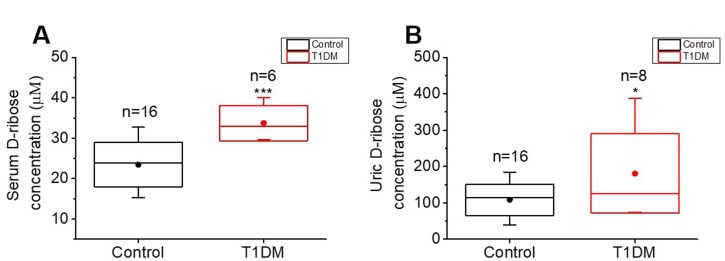
**Comparison of D-ribose levels between T1DM patients and normal participants.** Patients with T1DM (T1DM group) and age-matched normal participants (control group, n=16) were enrolled for determination of their D-ribose concentrations. D-ribose levels in serum (panel **A**) and urine (panel **B**) were measured by HPLC as previously described [17]. All values are shown as the mean ± S.E.M. *, *P* < 0.05; ***, *P* < 0.001.

To detect the D-ribose level in T1DM patients, twenty-four participants (8 T1DM patients and 16 age-matched participants without diabetes mellitus) were recruited for collection of fasting blood and morning urine. The summarized characteristics of the participants are shown in Supplementary Table 2. No differences were found in the age distributions between the T1DM and control groups (*P* = 0.8842), while FBG, HbA1c levels and BMI were significantly different between the two groups (*P* < 0.0001, *P* < 0.0001 and *P* = 0.0164, respectively). We measured the concentrations of D-ribose in urine and serum by high-performance liquid chromatography (HPLC). Both the serum and urine D-ribose levels of T1DM patients were significantly higher than those of controls (*P* = 0.0005 and *P* = 0.0285, respectively, Figure 5A, 5B), exhibiting the dysmetabolism of D-ribose in T1DM patients.

## DISCUSSION

T1DM results in long-term complications in the central nervous system, causing brain cellular dysfunction and cognitive deficits [40]. As reported in a recent study, T2DM patients suffer from D-ribose and D-glucose dysmetabolism [16]. In the present study, in addition to D-glucose, endogenous D-ribose was markedly increased in T1DM patients and STZ-induced T1DM rats, which also exhibited AGE accumulation and cognitive impairment accompanied with Tau hyperphosphorylation and neuronal death. Administration of BTMP decreased D-ribose levels and AGE accumulation and improved cognitive ability in T1DM rats. BTMP also reduced Tau hyperphosphorylation, and neuronal death. All these data indicated that D-ribose dysmetabolism is associated with cognitive impairment in T1DM rat and that the administration of BTMP can ameliorate the loss of neurons and cognitive impairment via regulation of D-ribose.

As a very active aldose, D-ribose exists in urine [16], serum [41] and cerebrospinal fluid (0.01 ~ 0.1 mM) [42]. D-Ribose increases AGE levels more significantly and rapidly than D-glucose does when the concentration ratio [D-ribose]/[D-glucose] is 1/50 [23]. AGE accumulation is one of the most important features in diabetes and its complications due to the high sugars levels [43]. According to the suggestion of the European Food Safety Authority, the maximum proposed levels is 10 g/day of D-ribose in food supplements for human adults and 3.6 g/kg bw per day of derived D-ribose for rat experiments [25]. The Panel considers that the effects observed in a subchronic toxicity study in Wistar rats could be the consequence of nutritional imbalances but that toxicological effects could not be ruled out [25]. In accordance with the Guidance for Industry, Center for Drug Evaluation and Research, U.S., the Conversion of Animal Doses to Human Equivalent Doses for rat and mouse in mg/kg are divided by a factor of 6.2 and 12.3, respectively. Administration of an approximate dose (3.2g/kg bw, once daily) of D-ribose has significant effects on mouse cognitive ability [27, 44]. D-ribose can also give rise to Tau hyperphosphorylation and Aβ-like deposition in brain tissue and cause endoplasmic reticulum stress, which is toxic to cells and results in apoptosis [27, 39, 45]. Administration of D-ribose can increase hepatic triglyceride and water intake and decrease body weight in SD rats [24]. Furthermore, formaldehyde was regarded as a risk factor for age-related cognitive impairment [46]. Intraperitoneal injection with D-ribose (3.2g/kg bw, once daily) leads to an increase in formaldehyde in the mouse brain [47]. These finding suggest that high dose D-ribose intake induces toxicity. A high level of D-ribose was also found in the urine of T2DM patients, suggesting the dysmetabolism of D-ribose in T2DM [16]. Therefore, speculation that D-ribose dysmetabolism is involved in type 1 diabetic encephalopathy and its pathogenesis is reasonable.

T1DM rats showed a low level of TKT in brain tissue accompanied by a high level of D-ribose and pathological features of diabetic encephalopathy. Because D-ribose can be converted from D-glucose through the PPP [48, 49], we measured G6PD and ribokinase. However, G6PD and ribokinase did not show a marked change in T1DM rats except for TKT. These results indicated that STZ-induced T1DM rats did not suffer from dysfunction in G6PD, a key enzyme in the D-glycolytic pathway. Many studies have shown that G6PD is upregulated [50] or downregulated [51] in STZ-induced DM rats. Epel demonstrated that the level of G6PD can be regulated by NADP, the critical factor in oxidative stress [52, 53]. Many studies have also shown that the expression of G6PD regulates the generation of NADPH to alleviate oxidative stress [54], suggesting that G6PD would induce dynamic changes in DM. Mendez et al. showed that neurons maintain the oxidation of G6PD through the PPP to sustain their antioxidant status [55]. In DM, apart from G6PD, TKT is also an important shunt key enzyme in the PPP [34]; thus, it makes sense that supplementation with BTMP, acting as a TKT activator [56], causes a reduction in oxidative stress and affects several anabolic reactions, which might also reduce the level of AGEs [57, 58]. BTMP regulates the level of TKT that is directly involved in D-ribose metabolism [59, 60]. In this studies, BTMP treatment upregulated TKT, decreased D-ribose levels and simultaneously rescued T1DM rats from diabetic-related encephalopathy but did not decrease D-glucose levels. These data indicated that dysmetabolism of D-ribose with a decline in TKT function was involved in cognitive impairment in T1DM rats under the experimental conditions. Other laboratories also observed dynamic changes of TKT activity in different tissues in diabetes [61, 62]. Consequently, TKT may be used as a potential drug target in the treatment of diabetic-related encephalopathy with high D-ribose levels.

BTMP gives a relatively wide range of actions on a number of cellular targets [58] such as treatment of inflammatory [63], peritoneal dialysis [64] and Tauopathy [65]. BTMP also plays a role in the metabolism of D-glucose [66]. BTMP also decreased oxidative stress and phosphorylation/activation of vascular endothelial growth receptor-2 [67] and Akt signaling [68]. According to Hammes and colleagues, BTMP activates TKT and prevents the activation of multiple pathways of hyperglycemic damage, such as the hexosamine pathway, the AGE formation pathway and the diacylglycerol-protein kinase C pathway, in diabetic animals [56]. Though activation of TKT through BTMP is downstream of the D-ribose pathway, which may not be direct evidence, the current work at least showed that a decrease in D-ribose levels could help the amelioration of cognitive impairment.

In clinical investigations, BTMP significantly improved the cognitive abilities of mild-to-moderate Alzheimer’s disease patients independently of brain amyloid accumulation [69]. In fact, BTMP is closely related to D-ribose metabolism. BTMP markedly ameliorates the impaired spatial cognitive ability of T1DM rats in the Y maze and Morris water maze. Administration of BTMP decreases D-ribose levels in the brain, blood and urine of T1DM rats. Chen and coworkers have indicated the correlation between D-ribose and the administration of BTMP in a ZDF rat animal model for diabetes [17]. Interestingly, urine D-ribose is negatively correlated with mini-mental state examination (MMSE) scores of patients with Alzheimer’s disease [70]. Currently, a clinical trial on the treatment of cognitive impairment in Alzheimer's disease (a pilot study) with BTMP has been started and performed by Gibson and Jordan in the Burke Neurological Institute (clinicaltrials.gov). Here, we would like to suggest that changes in D-ribose in blood and urine should be monitored and analysed in their clinical trials because BTMP can reduce D-ribose levels and ameliorate cognitive impairment in T1DM rats.

As mentioned above, neuronal death was observed in the hippocampal CA4/DG region of T1DM rats. Neuronal loss may deteriorate cognitive ability. CA4 is a subfield of the hippocampus that is adjacent to the DG subfield [71, 72]. The DG region, which is involved in long-term potentiation (LTP) and long-term memory, is associated with cognitive ability [73, 74]. Furthermore, the CA4/DG region in the brain controls anxiety-like behaviour and hippocampal neurogenesis and plasticity [75, 76]. Here, we also found that T1DM rats showed anxiety-like behaviour (Supplementary Figure 8). In previous studies, BTMP was also shown to counteract anxiety-like behaviour [77, 78]. Thus, determining whether there is anxiety in D-ribose-treated animals and whether D-ribose preferentially affects the CA4/DG region in the brain is worthwhile. Why the hippocampal CA4/DG region is more vulnerable to D-ribose dysmetabolism is also an important question. The connection of D-ribose with CA4/DG neuronal death and anxiety and the underlying mechanism need further investigation.

The current work suggests that dysregulated D-ribose acts as a novel metabolite in cognitive impairment in T1DM rats by triggering protein glycation, Tau hyperphosphorylation and neuronal loss. This viewpoint is based on the following observations. First, T1DM rats demonstrated high levels of D-ribose in the serum, urine and brain. Second, T1DM patients also showed high levels of D-ribose in the urine and serum. Third, the expression and activity levels of TKT in the brain and liver of T1DM rats were reduced, which affected D-ribose metabolism [60]. Fourth, gavage of BTMP as the activator upregulated the expression of TKT in the brain and liver and decreased the levels of D-ribose but not those of D-glucose. Fifth, administration of BTMP suppressed D-ribose levels and rescued cognitive impairment in T1DM rats in both the Y maze and Morris water maze assays, which confirmed the influence of D-ribose dysmetabolism. Sixth, in T1DM rats with cognitive impairment, AGE accumulation and Tau hyperphosphorylation in the hippocampus and cortex were closely related to D-ribose dysmetabolism. Seventh, amelioration of neuronal loss in hippocampal CA4/DG regions occurred with decreased D-ribose levels. Finally, on the basis of previous work in this laboratory, AGE accumulation, Tau hyperphosphorylation and cognitive impairment were observed in a D-ribose-induced mouse model [27, 79]. As described by other studies, both AGE [80] and Tau hyperphosphorylation [81] are associated with neuronal death [82] or loss [83, 84], which can cause hippocampal atrophy [85] and result in cognitive impairment [86, 87]. Therefore, D-ribose-induced neuronal loss may be an important contributor to cognitive impairment in T1DM rats.

In conclusion, STZ-induced T1DM rats had high levels of D-ribose in their brain, serum and urine in addition to D-glucose. TKT controlled D-ribose metabolism, and activation of TKT with BTMP decreased D-ribose levels, followed by a reduction in AGE formation, Tau hyperphosphorylation, neuronal death and cognitive impairment. Thus, dysmetabolism of D-ribose is considered a novel pathological features in rats with T1DM and its complications. T1DM patients also show high levels of D-ribose. However, further investigations should be conducted on T1DM pathologies and complications related to D-ribose.

## MATERIALS AND METHODS

### Animal treatments

Male SD rats (~8 weeks, weighing 180~200 g) were provided by Vital River Laboratory Animal Technology Co. Ltd. (China). The animals were housed in plastic cages measuring 45×30×26 cm (4 rats in each cage). Rats were maintained under standard laboratory conditions, i.e., a well-aerated room with alternating light and dark cycles of 12 h, and had access to food and water ad libitum. The handling of rats and experimental procedures were approved by the Animal Welfare and Research Ethics Committee of the Institute of Biophysics, Chinese Academy of Sciences (Permit Number: SYXK2016-32).

STZ (70 mg/kg bw, Sigma Aldrich, USA) was intraperitoneally injected into rats to induce hyperglycaemic conditions, as observed in diabetic patients (n = 30) [88]. Rats in the control group (n = 10) were injected with equivalent volumes of citrate buffer (pH 4.2-4.5). Diabetes was diagnosed when the FBG level of rats was higher than 11.1 mmol/L 3 days after STZ injection [89]. Behavioral tests were carried out, and rats were sacrificed in the 10^th^ week.

For BTMP administration, all rats were randomly divided into four groups: Control (n = 10), BTMP (n = 10), T1DM (n = 20), and T1DM+BTMP (n = 20). BTMP was dissolved in CMC (1% m/v) and administered at 300 mg/kg bw via gavage daily [90]. Rats in the control group received a single intraperitoneal injection of citrate buffer and daily CMC gavage. Rats in the BTMP group received a single intraperitoneal injection of saline solution and daily BTMP gavage. Rats in the T1DM group received a single intraperitoneal injection of STZ and daily CMC gavage. Rats in the T1DM+BTMP group received a single intraperitoneal injection of STZ and daily BTMP gavage. Behavioural tests were carried out, and rats were sacrificed in the 10^th^ week.

### Y maze

The Y maze we used was composed of three equally spaced arms (120°; 47 cm long × 46 cm wide × 16 cm high, Beijing ZSdichuang Science and Technology Development Co., Ltd, China). Activity in the Y maze was used to measure spontaneous alternation performance (working memory) and locomotor activity. The rats were placed in one of the arm compartments and allowed to move freely for 5 min. The sequence of arm entries was manually recorded. Alternation was defined as an entry into all three arms in consecutive choices. Spontaneous alternation percentage (alternation %) was defined as the ratio of the arm entry choices that differed from the previous two choices to the total choices. Then, the number of maximum spontaneous correct alternations was calculated as the total number of arms entered minus 2, and the percentage was calculated as correct alternations/maximum alternations × 100% [27, 91].

### Morris water maze

The Morris water maze (Beijing ZSdichuang Science and Technology Development Co., Ltd, China) test was performed as previously described [92]. The apparatus consisted of a circular water tank (150 cm in diameter and 60 cm in height) containing water (22 ± 2 °C) to a depth of 40 cm that was rendered opaque by adding black food dye. A platform (12 cm in diameter and 38 cm in height) was submerged 2 cm below the water surface and placed at the midpoint of one quadrant. Rats were exposed to a visual platform before they were exposed to a hidden platform. The visual platform was the same as the hidden platform, but a ‘flag’ that extends above the water surface by approximately 15 cm was mounted on the platform. Each rat had four trials per day with the visual platform test for four consecutive days. For the hidden platform test, each rat received four periods of training per day for five consecutive days. The latency to escape from the water maze (that is, finding the submerged escape platform based on the four different markers pasted on the middle of the cylinder wall of the four quadrants) was calculated for each trial. On day 6, a probe test was carried out by removing the platform and allowing each rat to swim freely for 60 sec. The time that rats spent swimming in the target quadrant (where the platform had been located during the hidden platform training) was measured. All data were recorded with a computerized video system [92].

### Open field test and elevated-plus maze

We performed elevated plus maze and open field tests as described [27, 93]. In the elevated plus maze, the percentage (%) of the time spent in the open arms, the number of open arm entries and head-dipping within 5 min was recorded. In the open field test, the percentage (%) of time spent in the center square, the number of center square entries, the number of stands and the number of grooming behaviors were considered indices of anxiety.

### Sample collection from animals

Urine samples were collected for D-ribose detection (every other week) when the experiment started. After completing the behavioral tests on week 10, all the rats were fully anaesthetized by using 10% chloral hydrate solution before being sacrificed. Then, the blood was collected as previously described [94] and centrifuged (4,000 rpm, 15 min, 20 °C). Serum was stored at -80 °C for different measurements. The hippocampus and cortex were also quickly dissected for subsequent Western blotting and D-ribose or fixed in 4% paraformaldehyde for Nissl staining. Liver was collected as described previously [95] for TKT assay.

### Grip strength measurement

A tension metre (Bioseb, France) was used to test the forelimb grip strength in rats as described by Tilson and colleagues [96]. Rats were held by their tails, and their front paws grasped the grid. Five grip force measurements were made, and the LCD screen of the tension metre automatically displayed the maximum tensile strength each time. The average of five measurements was taken to represent the forelimb grip strength.

### Measurements of body weight, FBG and physiological and biochemical indexes

The body weight and FBG concentration of each rat were recorded every other week when the experiment started. FBG was tested using a Roche ACCU-CHEK blood D-Glucose meter (Roche, USA). The levels of physiological and biochemical indexes (ALT, AST, CREA-J, BUM, TC, TG, insulin and C-peptide) were measured and supplied by the Fred Clinical Inspection Institution (China).

### Measurement of D-ribose by HPLC

D-ribose in the urine and serum was measured as previously described [16, 17]. Urine samples were centrifuged (12,000 rpm, 4 °C, 10 min), and serum samples were centrifuged (12,000 rpm, 4 °C, 10 min) after the precipitation of serum proteins by the addition of three volumes of acetonitrile. A 0.4 mL aliquot of the supernatant was mixed with 0.6 mL 4-(3-methyl-5-oxo-2-pyrazolin-1-yl) benzoic acid (MOPBA, final concentration 150 mM in 250 mM NaOH in 50% methanol-water solution, Sigma Aldrich, USA) and then heated in a 70 °C water bath for 90 min, followed by additional centrifugation (12,000 rpm, 4 °C, 10 min). The mixture was acidified by the addition of 150 μL 2 M HCl solution to precipitate the excess MOPBA, centrifuged (12,000 rpm, 4 °C, 10 min), and ultimately filtered through 0.22 μm membranes. Next, 20 μL of the solution was subjected to HPLC (LC-20A, Shimadzu, Japan) with an ultraviolet detector. Mobile phase A was 10 mM sodium 1-hexanesulfonate (Tokyo Chemical Industry, Japan), and mobile phase B was a 50% acetonitrile solution. The reference concentrations of D-ribose and D-glucose were determined according to the standard curves.

### Glycated serum protein (GSP) detection

The nitroblue tetrazolium (NBT) assay was used to detect GSP formation in serum samples [97]. The samples were mixed with NBT dye, and the absorbance of the samples was measured at 540 nm. The final content was calculated according to the manufacturer’s instructions (Nanjing Jiancheng, China). More details are provided in the instructions for specific experimental descriptions.

### Gel electrophoresis and Western blotting

The levels of AGE in the hippocampus and cortex were determined by Western blotting following standard protocols. The same method was used to analyse the expression of phosphorylated Tau at Ser396 (pS396) and AT8 (Ser199/202), nonphosphorylated Tau (Tau-1), and total Tau protein (Tau-5). The levels of TKT in the liver and brain were detected by Western blotting. β-Actin was used as a loading control. The antibodies used were as follows: anti-AGE monoclonal antibody (TransGenic, Japan), anti-Tau pSer396 polyclonal antibody (Invitrogen, USA), anti-Tau AT8 polyclonal antibody (Invitrogen, USA), anti-Tau-1 monoclonal antibody (Millipore, USA), anti-Tau-5 monoclonal antibody (Millipore, USA), anti-TKT antibody (Sigma, USA) and anti-β-actin monoclonal antibody (Sigma, USA).

### ELISA

The expression and activity levels of TKT, G6PD, PRPP and ribokinase in rat brains were quantified using ELISA kits in accordance with the manufacturer’s instructions. The ELISA kits used were as follows: TKT, G6PD, PRPP, and ribokinase expression kit (JiNingshiye, China), and TKT, G6PD, PRPP, and ribokinase enzyme activity kit (JiNingshiye, China).

### Nissl staining

Rat brains were processed for Nissl immunohistochemistry using standard protocols. Rat tissues were immersed in 4% paraformaldehyde for 48 h immediately after dissection. After fixation, the tissues were embedded in paraffin blocks. Sections (5 μm thick) were processed for Nissl staining according to the manufacturer’s instructions (Beyotime, China) [98]. The results were from ten independent samples for each group.

### Subject enrolment and sample collection

T1DM patients (n = 8) and normal subjects without diabetes (n = 16) were recruited from the Health Examination Center of the Affiliated Hospital of Southwest Medical University. The exclusion criteria for normal participators included diabetes, use of D-ribose as an energy supplement, or nephropathy or any other serious systemic diseases. None of the subjects had not undergone any surgery within 3 months. Their background characteristics are shown in Supplementary Table 2.

Morning urine and fasting blood samples were collected from the enrolled participants and stored in separate sealed sterile containers at -80 °C before measurements. The process strictly followed the regulations of the ethics committee of the Affiliated Hospital of Southwest Medical University (No. KY2017011), and written informed consent was obtained from all participants.

### Data analysis

We analysed all data using Origin 9.0 software (Originlab, USA). Significance differences between the T1DM group and the control group were calculated with two-sided unpaired Student’s t-tests. *P* values less than 0.05 were considered significant. All rescue experiments were performed using one-way ANOVA with a post hoc test. Differences with a probability level of 95% (*P* < 0.05) were considered significant.

## Supplementary Material

Supplementary Figures

Supplementary Tables
