# Supplementary material for: D-ribose is elevated in T1DM patients and can be involved in the onset of encephalopathy
Source: Aging (Albany NY). 2019 Jul 15;11(14):4943–69. doi: 10.18632/aging.102089 (PMC6682534; doi:10.18632/aging.102089)
Supplement: Supplementary Figures [file aging-11-102089-s002.pdf]

SUPPLEMENTARY FIGURES

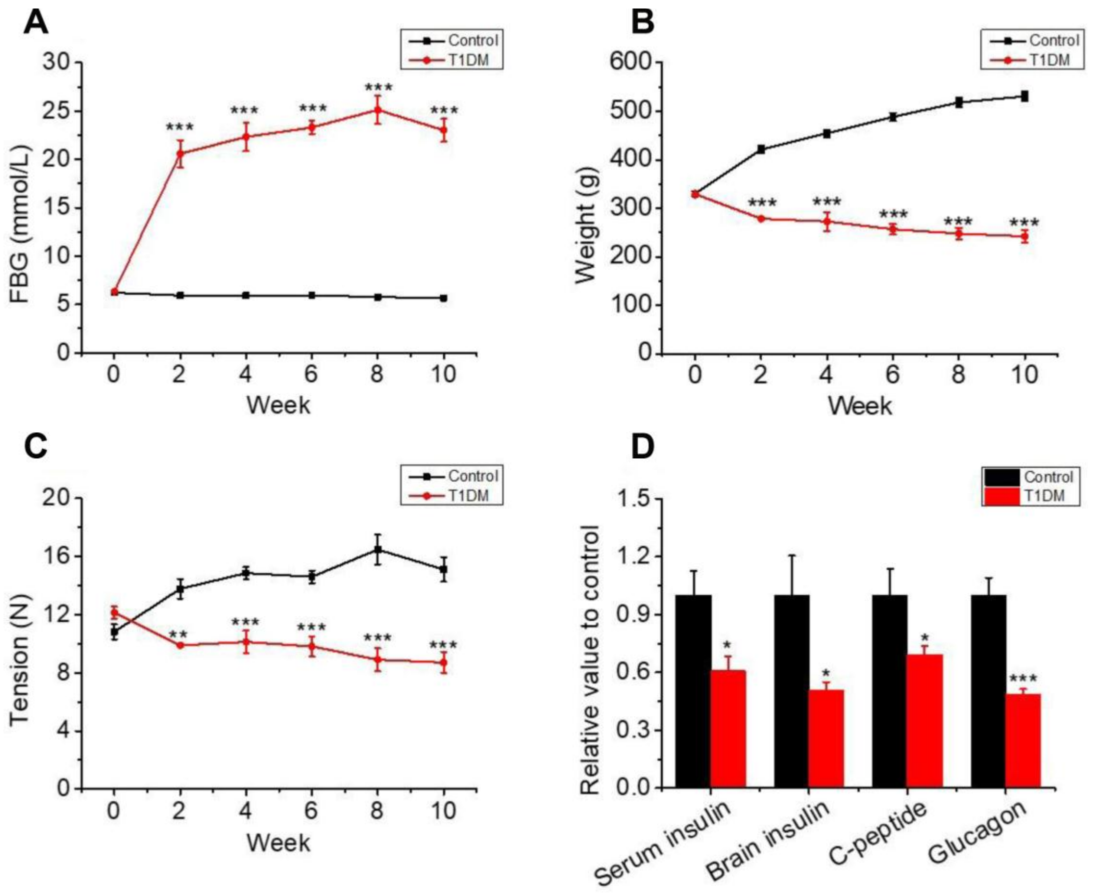

Supplementary Figure 1. Changes in FBG, weight, tension and insulin in T1DM rats. Male SD rats (6-8 weeks) were intraperitoneally injected with STZ (70 mg/kg bw, n=30) and maintained for 10 weeks. STZ (1%) was dissolved in citrate buffer at a pH of 4.4. Rats injected with citrate buffer were used as a control (n=10). Fasting blood glucose (FBG, panel **A**), body weight (panel **B**), and tension (panel **C**) of rats were monitored every other week. Rats were sacrificed after 10 weeks of acclimation, and serum insulin, brain insulin, serum C-peptide, and serum glucagon were measured (panel **D**). All values are expressed as the mean ± S.E.M. *, $P < 0.05$; **, $P < 0.01$; ***, $P < 0.001$.

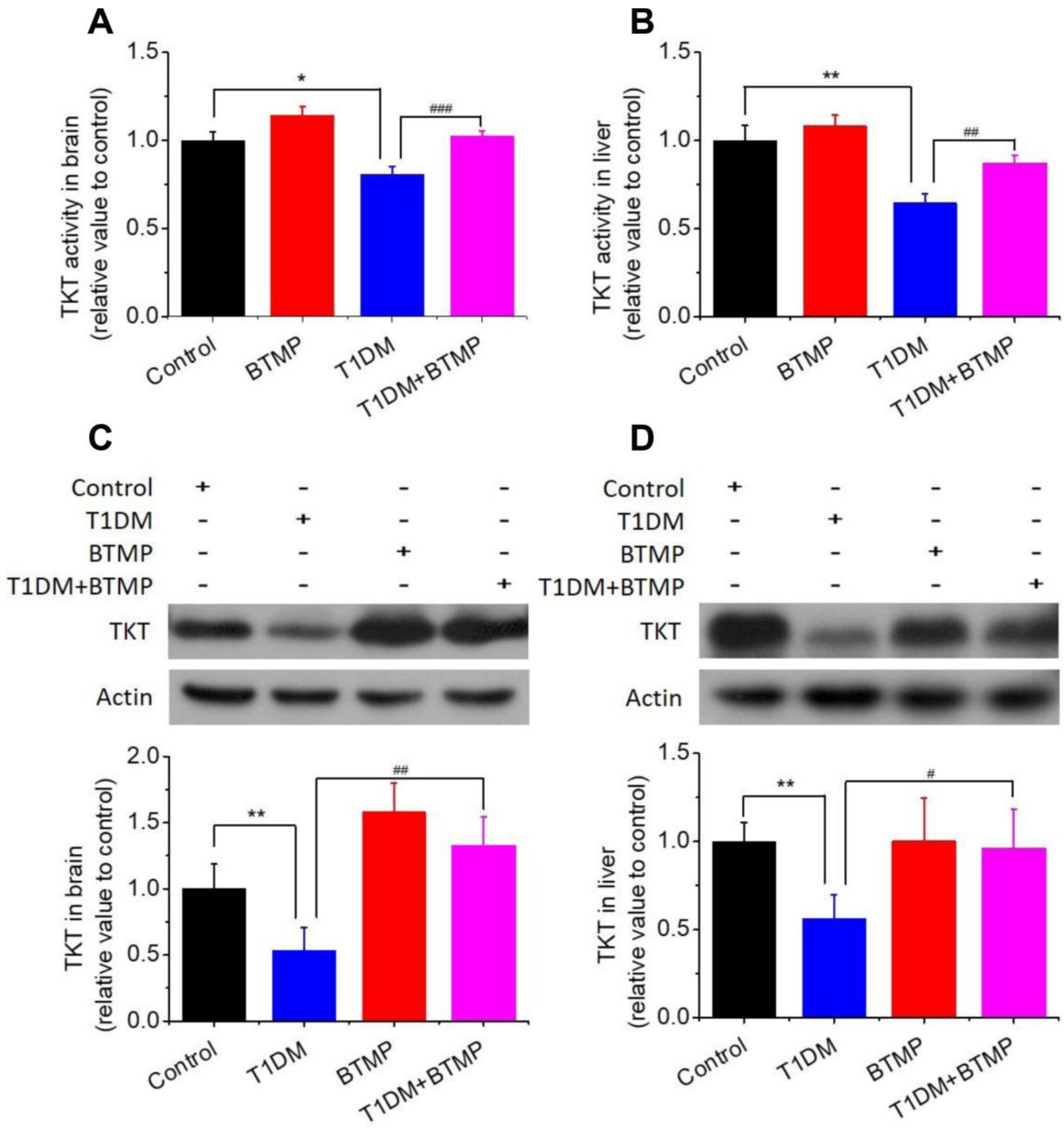

Supplementary Figure 2. The activity and Western blotting of BTMP-gavaged T1DM rats in the brain and liver. Conditions for the treatment were the same as those for Figure 2. The activity levels of transketolase (TKT) in the brain (panel **A**) and liver (panel **B**) were measured with ELISA kits. The numbers in the groups are control (n=10), BTMP (n=9), T1DM (n=15) and T1DM+BTMP (n=15). Western blotting of TKT using anti-TKT antibodies. β-Actin was used as a loading control. Quantifications were shown for the bands. The control value was set as 1.0. The number in each group is n=6. "*" compared to the control group. "#" represents the difference between the T1DM and T1DM+BTMP groups. All values are expressed as the mean ± S.E.M. *, $P < 0.05$; **, $P < 0.01$; #, $P < 0.05$; ##, $P < 0.01$; ###, $P < 0.001$.

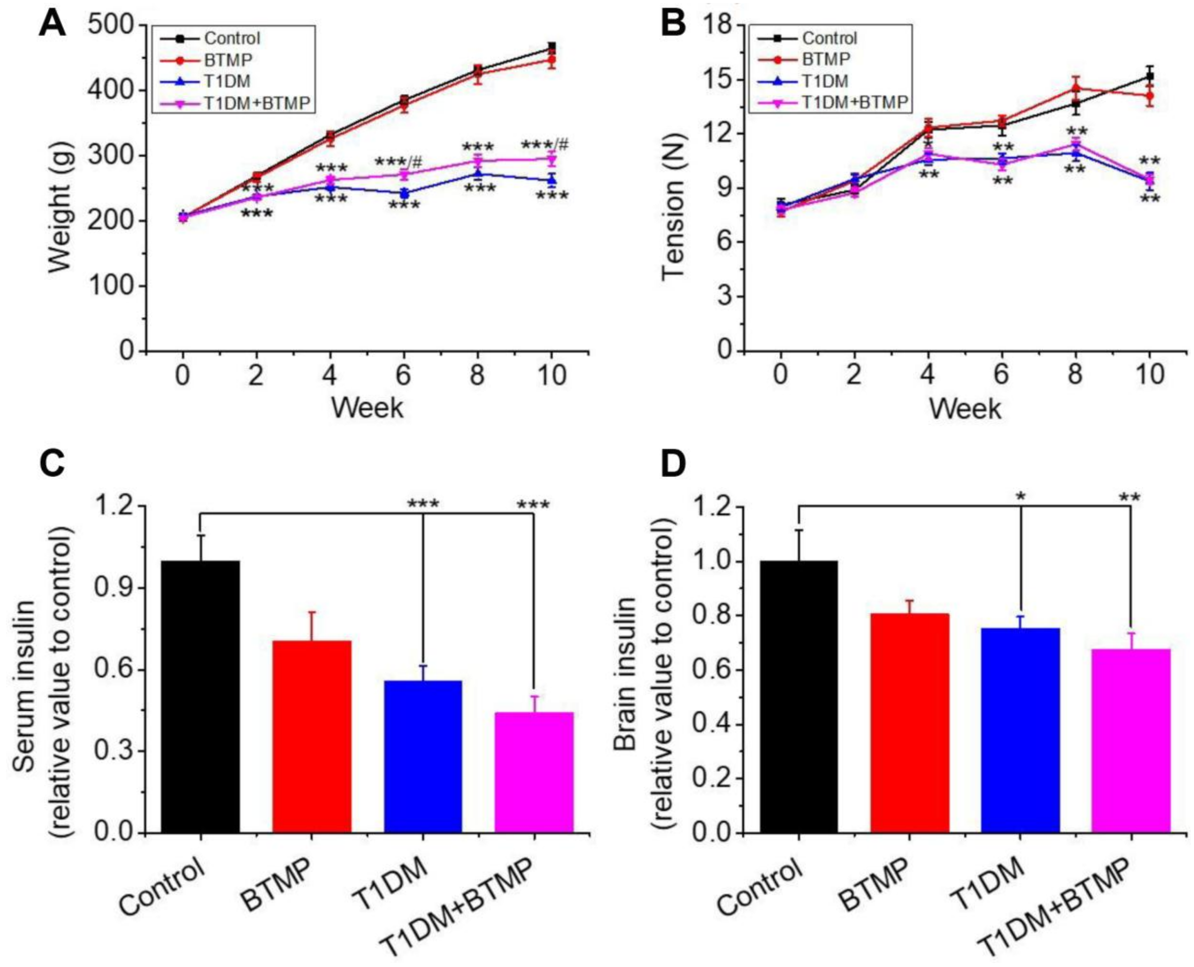

Supplementary Figure 3. Changes in body weight, tension and insulin in BTMP-gavaged T1DM rats. Conditions were the same as those in Figure 2. Body weights (panel **A**) and forepaw tension (panel **B**) of the four groups of rats (T1DM+benfotiamine (BTMP) (n=20), T1DM (n=20), BTMP (n=10) and Control (n=10)) were monitored every two weeks. After 10 weeks of acclimation, insulin in serum (panel **C**) and the brain (panel **D**) were determined. "*" compared to the control group. "#" represents the difference between the T1DM and T1DM+BTMP groups. All values are expressed as the mean ± S.E.M. *, $P < 0.05$; **, $P < 0.01$; ***, $P < 0.001$; #, $P < 0.05$.

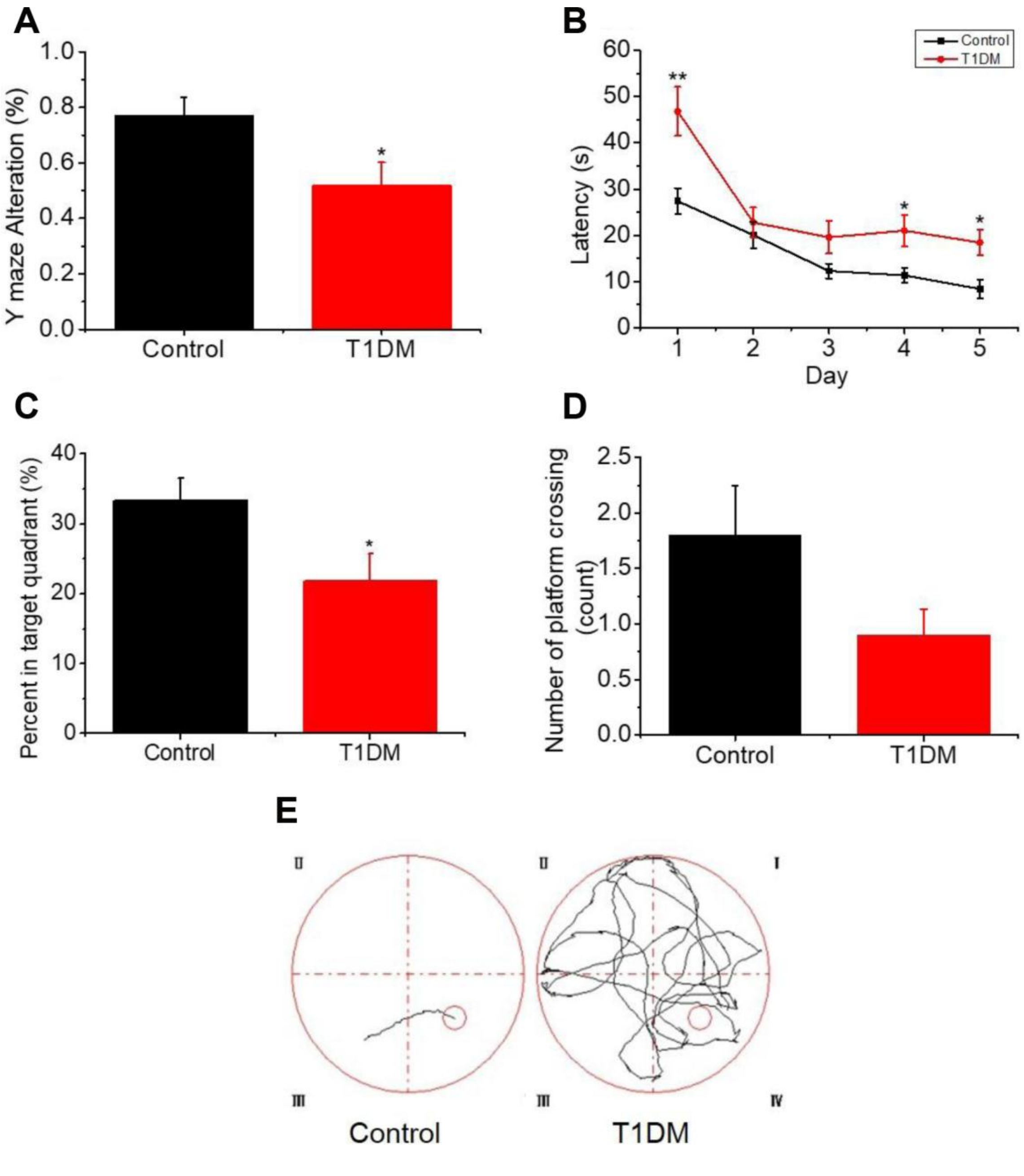

Supplementary Figure 4. Y maze and Morris water maze tests. Conditions for the treatment were the same as those for Supplementary Figure 1. The percentage of correct alternation in the Y maze was used to measure the exploration of a new environment (panel **A**). The time needed to find the hidden platform was recorded as escape latency for each of the five training days (panel **B**). The percentage of search time was recorded in the target quadrant from which the platform had been removed during the probe trial (panel **C**). The number of platform crossings in the probe trial (panel **D**) and the representative images of the performance path (panel **E**) are also shown. The numbers of those groups are control (n=10) and T1DM (n=13). All values are expressed as the mean ± S.E.M. *, $P < 0.05$; **, $P < 0.01$.

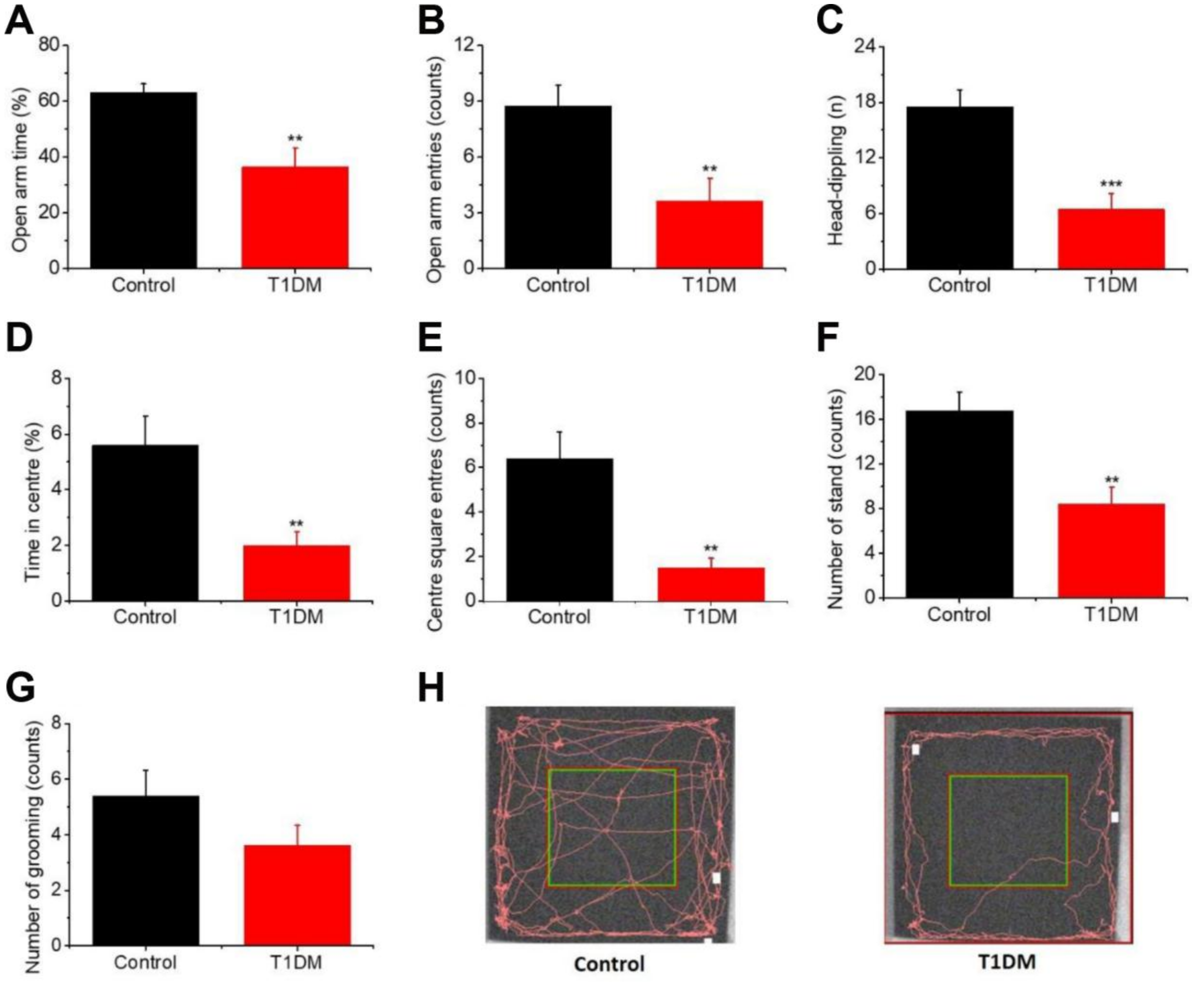

Supplementary Figure 5. Elevated plus maze and open field test. In the elevated plus-maze, the percentage of the time spent in the open arms (panel **A**), the number of open arm entries (panel **B**) and head-dipping (panel **C**) within 5 min was recorded. In an open field area, the percentage of time spent in the center square (panel **D**), the number of center square entries (panel **E**), number of stands (panel **F**) and number of grooming behaiors (panel **G**) are considered as an index of anxiety. Representative image of the performance path (panel **H**). "*" represents the difference between Control and T1DM groups. The P values are obtained from comparative analysis of the indicated group with the controls. The numbers in the groups are control (n=10) and T1DM (n=12). All values are expressed as the mean ± S.E.M. **, $p \leq 0.01$; ***, $p \leq 0.001$.

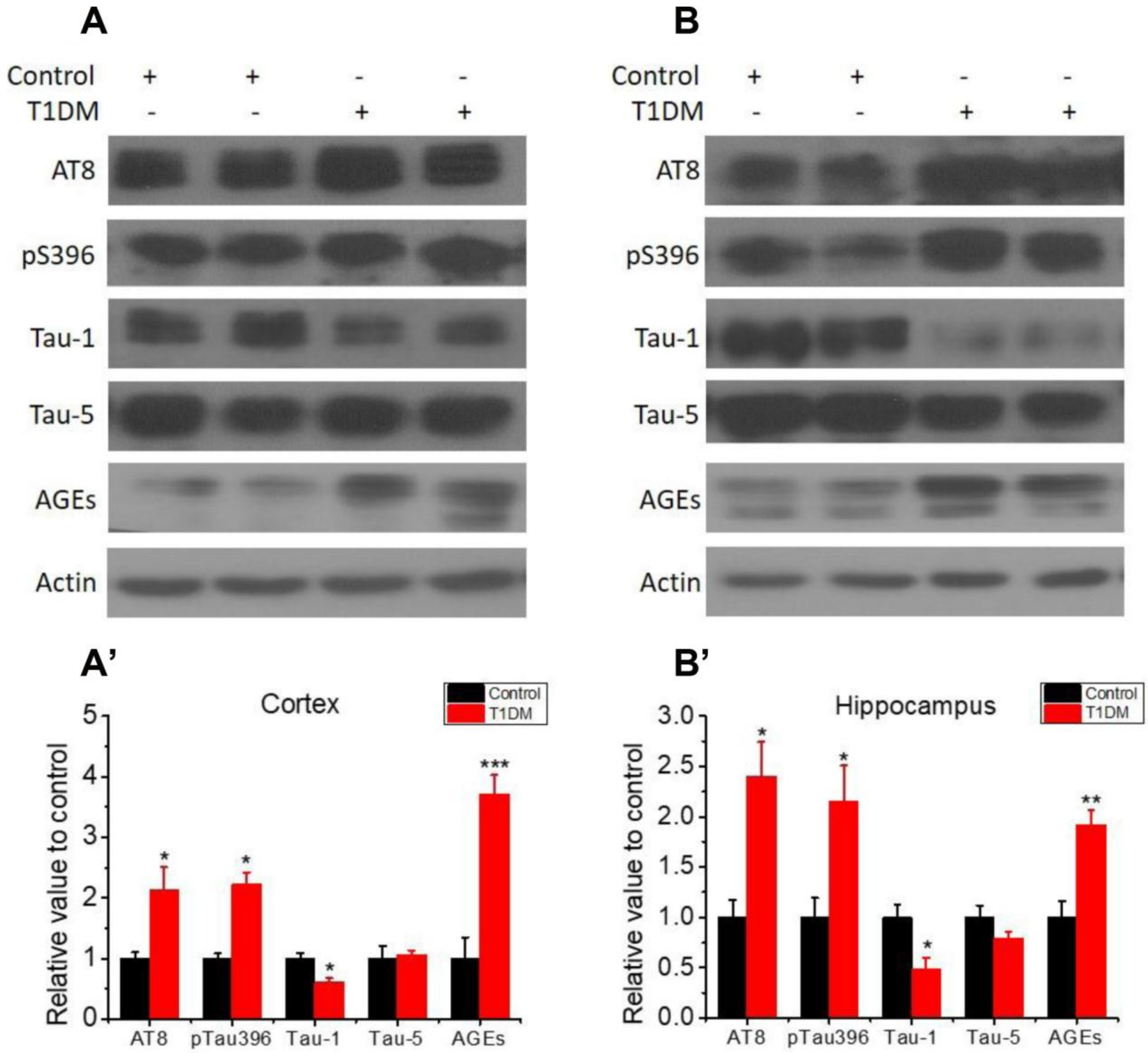

Supplementary Figure 6. Tau phosphorylation and AGE in the cortex and hippocampus of T1DM rats. Conditions for the treatment were the same as those for Figure 1 except phosphorylation of Tau and AGE in the cortex (panel **A**) and hippocampus (panel **B**) were detected by Western blotting using anti-pS396, anti-AT8, anti-Tau-1, anti-Tau-5 and anti-AGE antibodies. β-Actin was used as a loading control. Quantification is shown in panel **A'** and panel **B'**. The control value was set as 1.0. The phosphorylation levels were expressed as the ratio between phosphorylated-site and total Tau protein. The numbers in the groups are control (n=6) and T1DM (n=6). All values are expressed as the mean ± S.E.M. *, $P < 0.05$; **, $P < 0.01$; ***, $P < 0.001$.

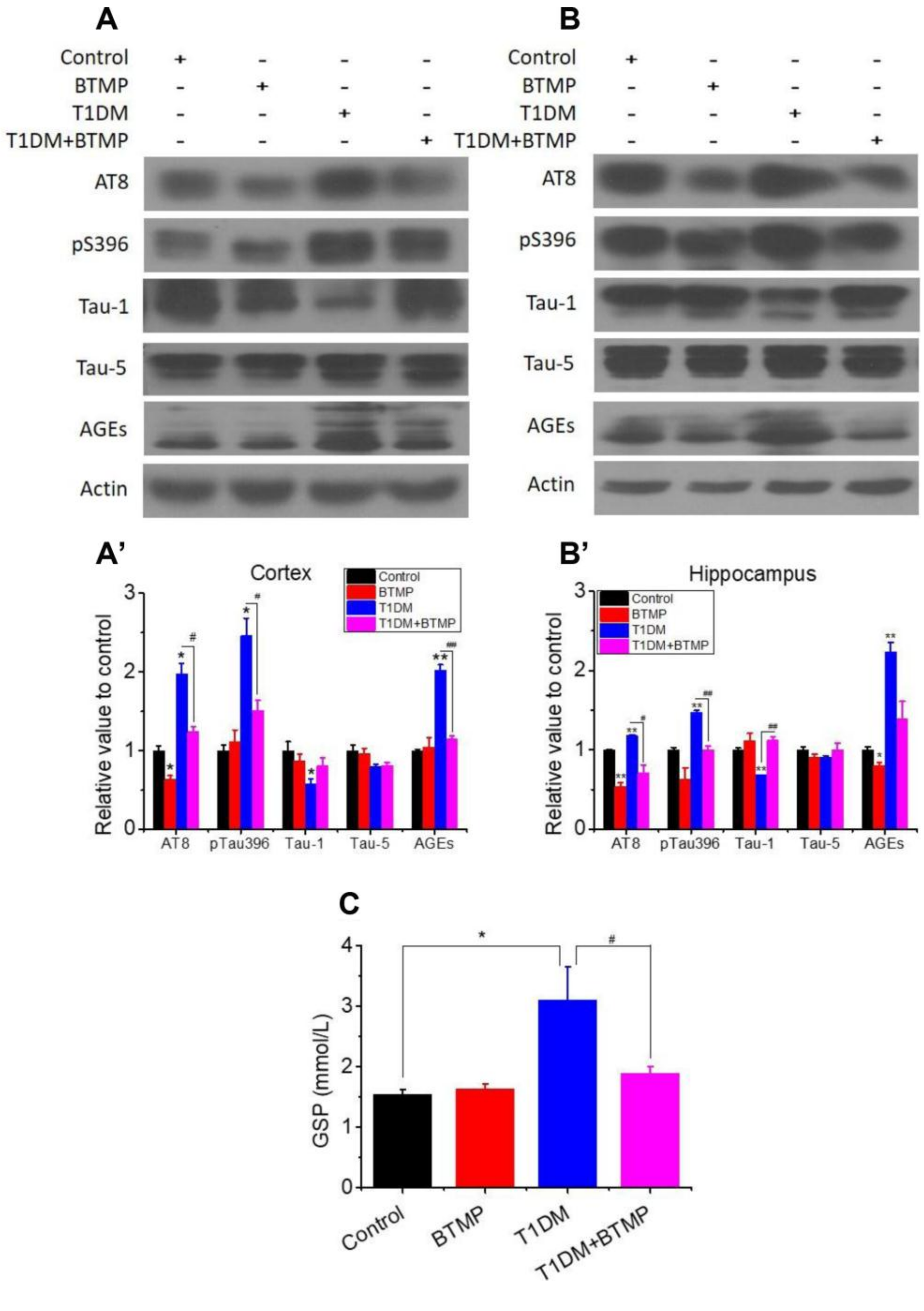

Supplementary Figure 7. Tau phosphorylation and AGE in the cortex and hippocampus of BTMP-gavaged T1DM rats. Conditions for the treatment were the same as those for Figure 2 except phosphorylation of Tau and AGE in the cortex (panel **A**) and hippocampus (panel **B**) were detected by Western blotting using anti-pS396, anti-AT8, anti-Tau-1 anti-Tau-5 and anti-AGE antibodies. β-Actin was used as a loading control. Quantifications are indicated in panel **A'** and panel **B'**. The control value was set as 1.0. The phosphorylation levels were expressed as the ratio between phosphorylated-sites and total Tau staining. The glycated serum protein level was detected using a GSP kit (panel **C**). "*" compared to the control group. "#" represents the difference between the T1DM and T1DM+benfotiamine (BTMP) groups. The numbers in the groups are control (n=6), BTMP (n=6), T1DM (n=6) and T1DM+BTMP (n=6). All values are expressed as the mean ± S.E.M. *, $P < 0.05$; **, $P < 0.01$; #, $P < 0.05$; ##, $P < 0.01$.

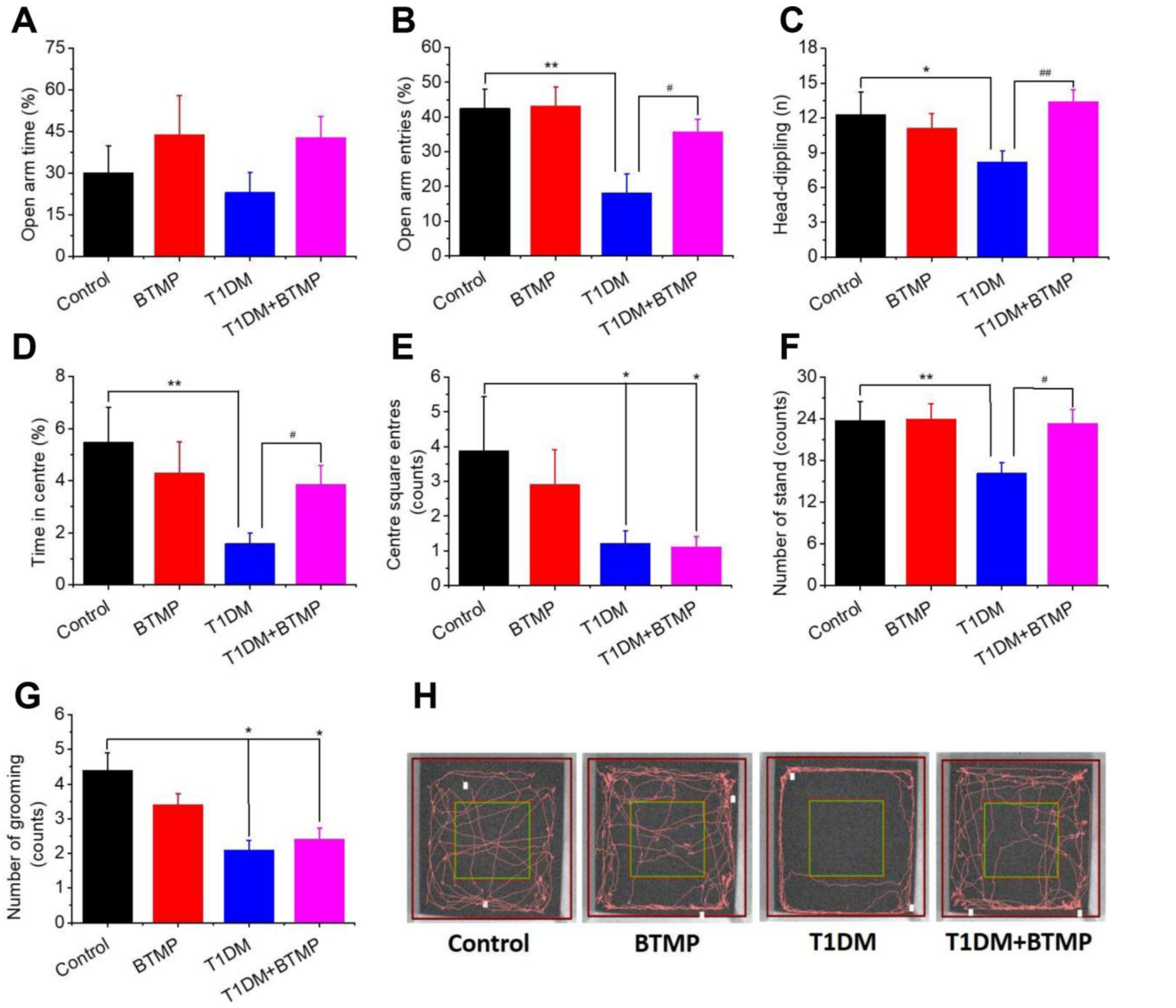

Supplementary Figure 8. Elevated plus maze and open field test. In the elevated plus-maze, the percentage of the time spent in the open arms (panel **A**), the percentage of open arm entries (panel **B**) and head-dipping (panel **C**) within 5 min was recorded. In an open field area, the percentage of time spent in the center square (panel **D**), the number of center square entries (panel **E**), number of stands (panel **F**) and number of grooming behaviors (panel **G**) are considered as an index of anxiety. Representative image of the performance path (panel **H**). "*" compared to the control group. "#" represents the difference between T1DM and T1DM+BTMP group. The P values are obtained from comparative analysis of the indicated group with the controls. The numbers in those groups are control (n=10), BTMP (n=10), T1DM (n=14) and T1DM+BTMP (n=15). All values are expressed as the mean ± S.E.M. *, $p \leq 0.05$; **, $p \leq 0.01$, #, $p < 0.05$; ##, $p < 0.01$.
