# Supplementary material for: D-ribose is elevated in T1DM patients and can be involved in the onset of encephalopathy
Source: Aging (Albany NY). 2019 Jul 15;11(14):4943–69. doi: 10.18632/aging.102089 (PMC6682534; doi:10.18632/aging.102089)
Supplement: Supplementary Tables [file aging-11-102089-s001.pdf]

SUPPLEMENTARY TABLES

Supplementary Table 1. Parameters of liver and kidney function.

	ALT(U/L)	AST(U/L)	CREA-J(umol/L)	BUN(mmol/L)	TC(mmol/L)	TG(mmol/L)
Control	47.6±2.4	291.3±10.9	88.6±3.1	4.7±0.1	1.6±0.1	1.4±0.1
BTMP	43.2±3.5	260.1±14.4	97.4±3.2	4.9±0.2	1.7±0.1	1.3±0.1
T1DM	124.9±18.8 **	320.9±15.9	103.1±4.0 *	7.3±0.4 ***	2.1±0.2 *	3.3±0.6 *
T1DM+BTMP	69.0±6.8 #	204.9±21.0 ##	103.8±2.6*	7.8±0.3 ***	1.9±0.1 **	1.2±0.1 ##

ALT, alanine transaminase; AST, aspartic transaminase; CREA-J, creatinine; BUN, blood urea nitrogen; TC, total cholesterol; TG, triglyceride. "*" compared to the control group. "#" represents the difference between the T1DM and T1DM+BTMP groups. All values are expressed as the mean ± S.E.M. *, P < 0.05; **, P < 0.01; ***, P < 0.001; #, P < 0.05; ##, P < 0.01.

Supplementary Table 2. Background characteristics of the participants.

	Age (year)	FBG (mM)	HbA1c (%)	BMI (kg/m^2)
Normal Subjects	50.63±1.93	4.66±0.10	5.71±0.05	23.93±0.72
T1DM Subjects	51.17±2.66	18.19±2.86	11.00±1.29	20.57±1.09

"FBG" is fasting blood glucose, and "HbA1c" is glycated hemoglobin. "BMI" is body mass index. All values are expressed as the mean ± S.E.M.
